# Transversus Thoracis Muscle Plane Block in Paediatric Patients Who Underwent Minimally Invasive Closure of Transthoracic Ventricular Septal Defect: A Retrospective Study

**DOI:** 10.1155/2023/3488552

**Published:** 2023-03-21

**Authors:** Qiong Ling, Shuhua Zhao, Yongyong Shi, Xiangyu Li, Ping Li, Gaofeng Zhao, Qianqian Zhu

**Affiliations:** ^1^Department of Anesthesiology, The Second Affiliated Hospital of Guangzhou University of Chinese Medicine, Guangzhou, China; ^2^Department of Anesthesiology, The Seventh Affiliated Hospital of Sun Yat-Sen University, Shenzhen, China

## Abstract

**Objective:**

Minimally invasive closure of transthoracic ventricular septal defect (VSD) has been widely used in paediatric patients. This retrospective study aimed to explore the use of transversus thoracis muscle plane block (TTMPB) in the minimally invasive closure of transthoracic VSD in paediatric patients.

**Methods:**

From September 28, 2017, to July 25, 2022, a total of 119 paediatric patients scheduled for minimally invasive transthoracic VSD closure were considered for inclusion.

**Results:**

In total, 110 patients were included in the final analysis. Perioperative fentanyl consumption of the TTMPB group was not different from that of the non-TTMPB group (5.90 ± 1.32 *μ*g/kg vs. 6.25 ± 1.74 *μ*g/kg, *p* = 0.473). Both the time to extubation and postanesthesia care unit (PACU) stay were significantly shorter in the TTMPB group than in the non-TTMPB group (10.94 ± 10.31 min vs. 35.03 ± 23.52 min for extubation, and 42.55 ± 16.83 min vs. 59.98 ± 27.94 min for PACU stay, both *p* < 0.001). Furthermore, the postoperative paediatric intensive care unit (PICU) stay in the TTMPB group was significantly shorter than in the non-TTMPB group (1.04 ± 0.28 d vs. 1.34 ± 1.05 d, *p* = 0.005). Multivariate analysis demonstrated that TTMPB was significantly associated with shorter time to extubation (*p* < 0.001) and PACU stay (*p* = 0.001) but not postoperative PICU stay (*p* = 0.094). *Discussion*. This study showed that TTMPB was a beneficial and safe regional anaesthesia technique for paediatric patients who underwent minimally invasive closure of transthoracic VSD, although prospective randomized controlled trials are needed to confirm the results.

## 1. Introduction

With the development of surgical technology and various devices, minimally invasive closure of the transthoracic ventricular septal defect (VSD) has been widely used in paediatric patients [1, 2]. Minimally invasive closure of transthoracic VSD was proven safe with excellent rates of closure [1, 3]. Although this surgery procedure leaves a small puncture on the anterior chest wall, postoperative pain management remains a challenge in paediatric patients [4, 5].

Pain management plays a critical role in the postoperative recovery of cardiac surgical patients [6]. Opioid-based postoperative pain management is the main strategy for cardiac surgery. However, opioids are associated with some well-known complications like nausea, vomiting, and respiratory depression. Multimodal opioid-sparing approaches, including regional anaesthesia techniques, show benefits and are encouraged [6, 7]. Ultrasound-guided transversus thoracis muscle plane block (TTMPB) is a relatively newly developed regional anaesthesia technique [8]. It has shown benefits in open cardiac surgery in paediatric patients [9, 10]. However, few studies have explored the efficacy of TTMPB in paediatric patients who underwent minimally invasive closure of the transthoracic VSD. Therefore, this retrospective study aimed to explore the use of TTMPB in the minimally invasive closure of transthoracic VSD in paediatric patients.

## 2. Methods

### 2.1. Ethical

This study was approved by the Ethics Committee of the Second Affiliated Hospital of the Guangzhou University of Chinese Medicine (chairperson: Prof. Yun Han, approval number: ZF2022-201-01) and was conducted in accordance with the Declaration of Helsinki. Due to the retrospective nature of the study and the use of anonymized data, the requirement for informed consent was waived by the ethics committee. It was registered in the Chinese Clinical Trial Registry at https://www.chictr.org (registration date: July 25, 2022; registration number: ChiCTR2200062147).

### 2.2. Sample Size

Considering the scarcity of studies on TTMPB in minimally invasive closure of transthoracic VSD and due to the retrospective nature of this study, all paediatric patients scheduled for minimally invasive closure of transthoracic VSD in the medical centre were considered.

From September 28, 2017, the minimally invasive closure of transthoracic VSD was started in our centre. The TTMPB will start to be performed in the surgery if there was no contradiction at our medical centre on December 23, 2020. Therefore, we divided the patients into two groups: those who underwent TTMPB were assigned to the TTMPB group, while the others were assigned to the non-TTMPB group.

### 2.3. Patients

From September 28, 2017 (when the surgery started in our centre) to July 25, 2022, a total of 119 paediatric patients scheduled for minimally invasive transthoracic VSD closure were considered for inclusion. The exclusion criteria were as follows: (1) transfer to open surgery; (2) transfer to the paediatric intensive care unit (PICU) with intubation after surgery; (3) transfer to interventional closure of VSD by percutaneous puncture; and (4) severe pulmonary hypertension.

Demographic data, such as sex, age, weight, duration of anaesthesia, duration of surgery, and time to extubation, were retrieved from medical records. Complications related to TTMPB were also retrieved from the records.

### 2.4. Anaesthesia Management

Anaesthesia was induced with intravenous fentanyl (2–3 *μ*g/kg), propofol (1.5–2.5 mg/kg), and vecuronium bromide (0.2 mg/kg) and maintained with propofol (3–12 mg/kg/h), vecuronium bromide (bolus doses), fentanyl (bolus doses), and sevoflurane.

Fifteen minutes before the end of the surgery, the patients were intravenously infused with fentanyl (0.5–1 *μ*g/kg) as an analgesic. The patients were then transferred to the postanesthesia care unit (PACU) at the end of surgery. The modified Aldrete scoring system was used as discharge criteria [11]. The patients were assessed every 15 min and transferred to the PICU for further observation if the scores were >12.

### 2.5. The TTMPB Procedure

**Figure 1:**
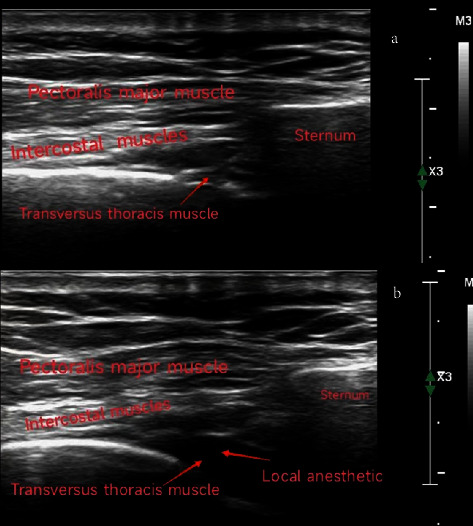
The ultrasound images in the TTMPB. (a) Ultrasound image before TTMPB; (b) ultrasound image after TTMPB. TTMPB: transversus thoracis muscle plane block.

A linear probe (L11-3, iE33, Philips, The Netherlands) was used to guide the procedure after anaesthesia induction and intubation. The probe was inserted into a sterile sheath and positioned one centimetre lateral to the sternum in a parasagittal manner for the purpose of counting ribs. At the intercostal space between the fourth and fifth ribs beside the edge of the sternum, a 20G 50 mm block needle was placed using the in-plane technique. When the tip of the needle reached the interfacial plane between the transverse thoracic and inner intercostal muscles (Figure 1(a)), 1 ml of saline water was injected to confirm the location through hydrodissection. Subsequently, 1.5 mg/kg (0.30%) ropivacaine was recommended to be injected on each side (no more than 150 mg) in our medical centre (Figure 1(b)). The TTMPB procedure was performed before the start of the surgery.

### 2.6. The Puncture of Surgery

After all the anaesthesia-related procedures were completed, the child was in the supine position. The position, size, and edges of the VSD and aortic valve were evaluated by transoesophageal echocardiography. A 2–4 cm incision was located in front of the lower end of the sternum for perimembranous VSD or in front of the middle sternum for subarterial VSD. In perimembranous VSD, the mediastinum was entered through the lower part of the sternum. In subarterial VSD, the skin incision was pulled to the left, and the mediastinum was entered through the third intercostal space on the left edge of the sternum.

### 2.7. Outcomes and Definition

The perioperative opioid consumption and the postoperative 24 h rescue analgesia were calculated. The time to extubation, PACU stay, and postoperative PICU stay were also retrieved from medical records.

The perioperative opioids were all the opioids consumed from the anaesthesia induction to the end of surgery. The postoperative rescue analgesia was calculated from the end of surgery.

### 2.8. Statistical Analysis

Qualitative data are presented as percentage/composition ratios. Quantitative data are presented as the mean ± standard deviation. The Kolmogorov−Smirnov test was used to test the normality of the quantitative data. The Levene's test was used to test the equality of variance. Depending on the distribution and equality, an independent *t*-test or Mann−Whitney test was used to compare differences. Multiple linear regression was used to do the multivariate analysis. A general linear model (GLM) was also used to assess differences in heart rate and blood pressure between the TTMPB and non-TTMPB groups at each time point. The response variable was the difference in the heart rate and blood pressure variation with two factors: group (the intervention) and time (before and after TTMPB). The interaction (group × time) was evaluated to determine if the effects were different between TTMPB and non-TTMPB over time. Two-tailed*p* values of <0.05 were considered significant. Statistical analyses were performed with SPSS software (version 22.0; IBM Corporation, Armonk, NY).

## 3. Results

### 3.1. Baseline Characteristics

A total of 110 patients were included in the final analysis after excluding five patients who were transferred to open surgery and four to the ICU without extubation because of intraoperative respiratory failure. There were 51 and 59 patients in the TTMPB and non-TTMPB groups, respectively. The inclusion and exclusion are shown in the flow chart.
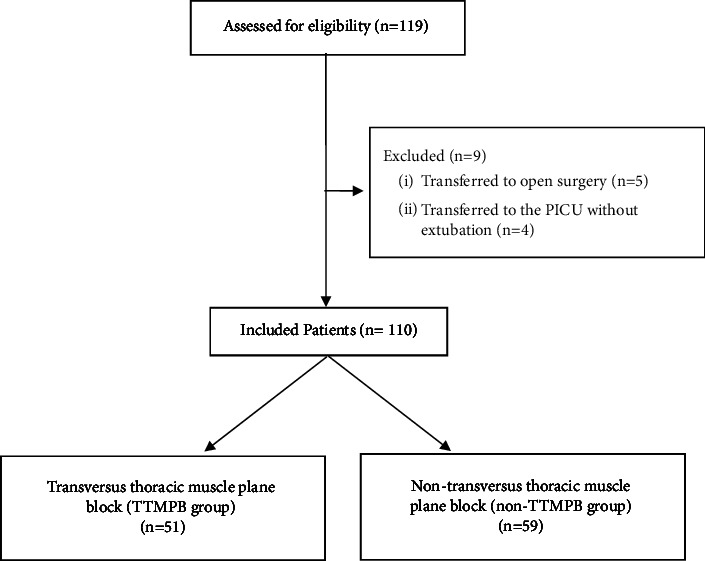


### 3.2. Results of Univariate Analysis

The age of patients in the non-TTMPB group was significantly younger than that of the TTMPB group (26.47 ± 26.49 months vs. 40.82 ± 33.00 months, *p* = 0.001, Table 1). The distribution of sex and American Society of Anaesthesiologists (ASA) physical status was not statistically different between the two groups.

Perioperative fentanyl consumption of the TTMPB group was not different from that of the non-TTMPB group (5.90 ± 1.32 *μ*g/kg vs. 6.25 ± 1.74 *μ*g/kg, *p* = 0.473, Table 1). Both the time to extubation and PACU stay were significantly shorter in the TTMPB group than in the non-TTMPB group (10.94 ± 10.31 min vs. 35.03 ± 23.52 min for extubation, and 42.55 ± 16.83 min vs. 59.98 ± 27.94 min for PACU stay, both *p* < 0.001, Table 1).

Furthermore, the postoperative PICU stay in the TTMPB group was significantly shorter than in the non-TTMPB group (1.04 ± 0.28 days vs. 1.34 ± 1.05 days, *p* = 0.005, Table 1).

Regarding the patients who received rescue analgesia 24 h postoperatively, there were no significant differences between the two groups (6.8% vs. 5.9%, *p* = 0.847, Table 1).

### 3.3. Results of Multivariate Analysis

The significant differences in age between the two groups might affect the results, including the time to extubation, PACU, and the postoperative PICU stay. Therefore, we did multivariate analysis through multiple linear regression. The results demonstrated that TTMPB was significantly associated with less time to extubation (*B* = −22.40, *p* < 0.001), PACU (*B* = −15.07, *p* = 0.001), but not postoperative PICU stay (*B* = −0.27, *p* = 0.094).

### 3.4. Results of Repeated Measurements

**Figure 2:**
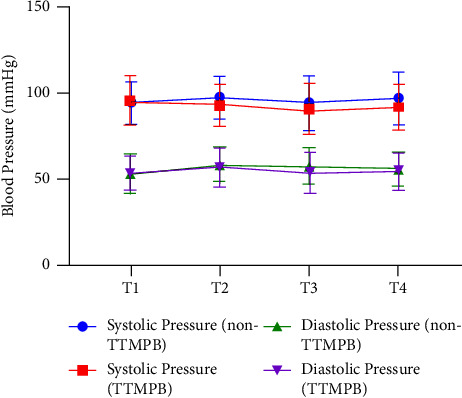
Blood pressure at different time points. T1, 10 min before TTMPB; T2, skin incision; T3, 1 h after surgery; T4, end of surgery. For systolic pressure: *p* = 0.034 over time change, *p* = 0.063 between the two groups, *p* = 0.549 for interaction; for diastolic pressure: *p* = 0.018 over time change, *p* = 0.065 between the two groups, *p* = 0.915 for interaction; TTMPB: transversus thoracis muscle plane block.

**Figure 3:**
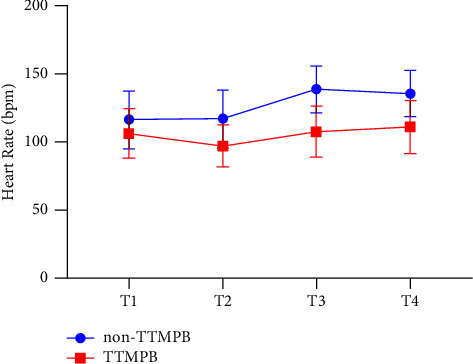
The heart rate of different time points. T1, 10 min before TTMPB; T2, skin incision; T3, 1 h after surgery; T4, end of surgery. *p* < 0.001 over time change, *p* < 0.001 between the two groups, *p* < 0.001 for interaction. TTMPB: transversus thoracis muscle plane block.

The blood pressure changed over time (*p* = 0.034 for systolic pressure, *p* = 0.018 for diastolic pressure; Figure 2), there were no significant differences between the TTMPB and non-TTMPB groups through GLM analysis (*p* = 0.063 for systolic pressure, *p* = 0.065 for diastolic pressure; Figure 2). However, the heart rate changed over time with the difference between the TTMPB and non-TTMPB (all *p* < 0.001, Figure 3).

## 4. Discussion

This study showed that TTMPB could significantly shorten the time to extubation and PACU stay in paediatric patients who underwent minimally invasive closure of the transthoracic VSD. The heart rate was more stable in the TTMPB group than in the non-TTMPB group.

TTMPB could provide satisfactory pain control because the asteria chest wall was blocked for sensory block distribution between T2-T6 [12]. A canine cadaver study also suggested that a single injection of TTMPB under ultrasound guidance could provide staining of three to four intercostal nerves [13]. Therefore, TTMPB has been found to be effective in several types of pain management, including post-thoracotomy pain syndrome and breast surgery [14, 15]. Furthermore, because TTMPB is superficial and ultrasound-guided, it is safe and easy to perform [16]. A previous study demonstrated that only a few self-limiting complications occurred in ultrasound-guided TTMPB, though future studies are required to confirm the results [17]. Compared to paravertebral and epidural analgesia, which are used for anterior chest wall analgesia and require lateral positioning, TTMPB is performed in the supine position. The unchanged position after anaesthesia induction might shorten the total anaesthesia time and provide hemodynamic stability. The present results demonstrated that heart rate was more stable in the TTMPB group than in the non-TTMPB group. Based on the block range of the anterior chest wall, the application and effects of TTMPB in open cardiac surgery with a median sternotomy were explored [18]. In comparison with open cardiac surgery, the present study demonstrated that TTMPB also showed beneficial effects in the minimally invasive closure of transthoracic VSD.

VSD is one of the most common congenital heart diseases in paediatric patients. With the advancement of transcatheter techniques and surgical development, minimally invasive closure of transthoracic VSD has been widely used in China [1, 19]. Although this minimally invasive procedure leaves a smaller puncture on the chest surface than the conventional median sternotomy incision, postoperative recovery remains challenging. As a newly developed regional anaesthesia technique, TTMPB was found to provide good perioperative analgesia to promote postoperative recovery in both adult and paediatric open cardiac surgery [9, 20, 21]. It has been shown that in children undergoing cardiac surgery through a median sternotomy, TTMPB can significantly decrease perioperative fentanyl consumption and reduce postoperative pain intensity [22]. Considering paediatric patients who undergo minimally invasive transthoracic VSD closure, the present study also suggested that TTMPB could significantly shorten the time to extubation and the PACU stay. However, TTMPB did not reduce the postoperative 24 h rescue analgesia requirement. It might be attributed to the limited duration of action of local anaesthetics.

As an important component of multimodal anaesthesia in cardiac surgery, regional anaesthesia has been used widely in cardiac surgery [23]. However, regional anaesthesia techniques showed limited impact on major clinical outcomes, though they can effectively treat pain [24, 25]. With the development of minimally invasive cardiac surgery, the incisions also move toward being smaller. The multimodal anaesthetic techniques which help with pain control and postoperative recovery in minimally invasive cardiac surgery [26]. To the best of our knowledge, this study fills the gap in exploring TTMPB in paediatric patients who undergo minimally invasive closure of transthoracic VSD.

However, there are several limitations to this study. First, it was a retrospective study that included a limited size of paediatric patients. The sample number in previous studies that explored TTMPB performed in open cardiac surgery was no more than 100 [18, 22]. Demographic characteristics were not comparable between the TTMPB and non-TTMPB groups. Though multivariate analysis was used, the results need to be confirmed in future randomized controlled trials. Second, TTMPB was performed under general anaesthesia, and dermatomal block could not be assessed. Although all TTMPB were performed under ultrasonic guidance by the same anesthesiologist, the actual block range of the intercostal nerves was unknown in the present study. Third, patient-controlled analgesia was not used for minimally invasive closure of the transthoracic VSD in our medical centre. Fourth, this study only analysed the postoperative 24 h period of rescue analgesia but not pain scores due to its retrospective nature and the fact that the included patients were paediatric. Moreover, the pain scores in postoperative periods, including PACU and PICU stays, were also lacking. Therefore, the effects of TTMPB on pain cannot be generalisable beyond 24 h.

In conclusion, this retrospective study showed that TTMPB was a beneficial and safe regional anaesthesia technique for paediatric patients who underwent minimally invasive transcatheter device closure of the VSD, although prospective randomized controlled trials are needed to confirm the results.

## Figures and Tables

**Table 1:** Characteristics, procedures, and perioperative data.

Group	Non-TTMPB (59)	TTMPB (51)	*p*
Age (m)	26.47 ± 26.49	40.82 ± 33.00	0.001
Male	29 (49.2%)	24 (47.1%)	0.827
BMI (kg/m^2^)	15.30 ± 1.51	15.52 ± 1.93	0.443
ASA grade			1.000
I-II	57 (96.6%)	50 (98.0%)	
III	2 (3.4%)	1 (2.0%)	
Anaesthesia time (min)	133.86 ± 41.53	139.88 ± 26.04	0.023
Operation time (min)	61.53 ± 36.12	64.10 ± 21.60	0.053
Fentanyl (*μ*g/kg)	6.25 ± 1.74	5.90 ± 1.32	0.473
Postoperative			
Time to extubation (min)	35.03 ± 23.52	10.94 ± 10.31	<0.001
PACU stay (min)	59.98 ± 27.94	42.55 ± 16.83	<0.001
Postoperative PICU stay (day)	1.34 ± 1.05	1.04 ± 0.28	0.005
Postoperative 24 h rescue analgesia	4 (6.8%)	3 (5.9%)	0.847

BMI: body mass index, ASA: American Society of Anesthesiologists, PACU: postanesthesia care unit, PICU: paediatric intensive care unit.

## Data Availability

The datasets used and analysed during the current study are available from the corresponding author on reasonable request.

## Abbreviations

VSD: Ventricular septal defect
TTMPB: Transversus thoracic muscle plane block
ICU: Intensive care unit
PACU: Postanesthesia care unit
PICU: Paediatric intensive care unit
GLM: General linear model.
